# A biphasic multilayer computational model of human skin

**DOI:** 10.1007/s10237-021-01424-w

**Published:** 2021-02-10

**Authors:** David Sachs, Adam Wahlsten, Sebastian Kozerke, Gaetana Restivo, Edoardo Mazza

**Affiliations:** 1 ETH Zurich, Institute for Mechanical Systems, Zürich, Switzerland; 2 University and ETH Zurich, Institute for Biomedical Engineering, Zürich, Switzerland; 3 Department of Dermatology, University Hospital Zürich, Zürich, Switzerland; 4 EMPA, Swiss Federal Laboratories for Materials Science and Technology, Experimental Continuum Mechanics, Dübendorf, Switzerland

**Keywords:** Human skin, Hyperelasticity, Poroelastic soft tissue, Inverse analysis, Biphasic material

## Abstract

The present study investigates the layer-specific mechanical behavior of human skin. Motivated by skin’s histology, a biphasic model is proposed which differentiates between epidermis, papillary and reticular dermis, and hypodermis. Inverse analysis of ex vivo tensile and in vivo suction experiments yields mechanical parameters for each layer and predicts a stiff reticular dermis and successively softer papillary dermis, epidermis and hypodermis. Layer-specific analysis of simulations underlines the dominating role of the reticular dermis in tensile loading. Furthermore, it shows that the observed out-of-plane deflection in ex vivo tensile tests is a direct consequence of the layered structure of skin. In in vivo suction experiments, the softer upper layers strongly influence the mechanical response, whose dissipative part is determined by interstitial fluid redistribution within the tissue. Magnetic resonance imaging-based visualization of skin deformation in suction experiments confirms the deformation pattern predicted by the multilayer model, showing a consistent decrease in dermal thickness for large probe opening diameters.

**Supplementary Information:**

The online version supplementary material available at 10.1007/s10237-021-01424-w.

## Introduction

Understanding and correctly predicting the mechanical properties of human skin are essential for medical applications. Planning of reconstructive surgery (Mollemans et al. 2007; Beldie et al. 2010; Lee et al. 2018), wound healing (Buganza Tepole and Kuhl 2016; Evans et al. 2013; Barnes et al. 2018; Chen et al. 2019) and growth through “expanders” (Zöllner et al. 2012, 2013; Buganza Tepole et al. 2011) depend on deformations as well as the force distribution within the skin. Investigating and unveiling the underlying mechanical and mechanobiological processes require accurate models of the skin’s response under diverse conditions of mechanical loading.

In order to characterize the mechanical properties of skin in vivo, several methods were proposed such as suction (Müller et al. 2018; Diridollou et al. 1998, 2000; Barbarino et al. 2011), indentation (Virén et al. 2018; Abellan et al. 2013; Iivarinen et al. 2014) and in-situ tension (Flynn et al. 2011; Bhushan et al. 2010). Uniaxial tension (Wahlsten et al. 2019; Ní Annaidh et al. 2012), biaxial tension (Tonge et al. 2013) and shear experiments (Soetens et al. 2018; Lamers et al. 2013; Geerligs et al. 2011) were performed to determine mechanical properties of skin ex vivo. All data show a pronounced J-shaped stress–strain response, typical for biological tissues. The experimental observations were rationalized using several nonlinear material models, as reviewed by Limbert (2017), Benítez and Montáns (2017) and Joodaki and Panzer (2018). Model formulations include the hyperelastic isotropic neo-Hookean (Flynn and McCormack 2008; Delalleau et al. 2008) or Arruda–Boyce models (Bischoff et al. 2000), the an- isotropic Holzapfel–Gasser–Ogden model (Ní Annaidh et al. 2012) and the viscoelastic Rubin–Bodner model (Weickenmeier et al. 2014; Wahlsten et al. 2019). Most previous approaches model the skin as a single-layer, mono-phasic, incompressible material. It’s histological composition (Montagna and Parakkal 1974) and recent uniaxial mechanical observations (Wahlsten et al. 2019) suggest, however, a multilayer and multi-phase representation. Epidermis, papillary dermis and reticular dermis show a clear difference in cellular composition as well as in collagen content and structure (Ruth and Freinkel 2001), which is expected to result in markedly different mechanical properties. Indeed, multilayer models as well as attempts to characterize single layers of skin have been undertaken. Hendriks et al. (2006) used suction to differentiate between the reticular dermis and the upper layer, composed of epidermis and papillary dermis. They concluded that the upper layer is several orders of magnitude softer. Crichton et al. (2011) applied nanoindentation on dermis and epidermis samples of murine skin to show that dermis shows the stiffest instantaneous response, while force–relaxation were most pronounced in epidermis. Soetens et al. (2018) performed ex vivo shear experiments and divided the skin geometrically into ten layers. Remarkably, they found the highest shear stiffness to be within the papillary dermis. Further, existing multi-phasic models highlight the importance of interstitial fluid mobility for the mechanical response of skin (Wahlsten et al. 2019; Oomens et al. 1987; Abellan et al. 2013, 2014; Oftadeh et al. 2018).

The objective of the present work is to develop a mechanical model of skin’s layered structure. We first introduce a multilayered biphasic model of skin, based on its histological structure. Second, material parameters are determined for each layer based on a wide range of ex vivo and in vivo experimental data. Finally, we investigate and discuss layer-specific mechanical properties and local changes with physiological deformation in-vivo.

## Skin as a multilayered material

**Fig. 1:**
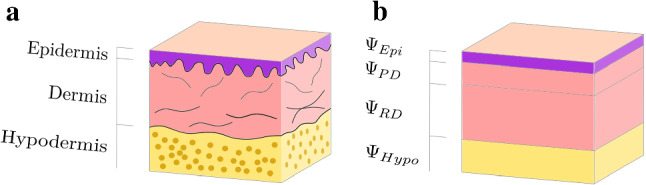
Layered model, (**b**), differentiates epidermis, papillary and reticular dermis and hypodermis, based on skin’s histology (**a**)

Human skin consists of several layers, of which the main three are epidermis, dermis and hypodermis, as shown in Fig. 1 (Ruth and Freinkel 2001; Montagna and Parakkal 1974).

### Layers histology

The epidermis is the outermost layer of the skin. It consists mainly of keratinocytes, melanocytes, Langerhans cells and Merkel cells. Starting from the basal layer, keratinocytes migrate upwards during their lifetime and undergo differentiation. Because of this process, the epidermis is often divided into a living and a cornified cell region, with the stratum corneum building its uppermost layer. At the lower end, the epidermis is connected to the dermis by the dermal–epidermal junction, a complex network of interconnecting proteins (Ruth and Freinkel 2001; Burgeson and Christiano 1997). Hemidesmosomes and anchoring fibrils within the dermal–epidermal junction firmly connect it to epidermis and dermis, respectively (Briggaman and Wheeler 1975; Turcan and Jonkman 2015; Rohrbach and Timpl 1993).

The dermis, in contrast with the epidermis, contains fewer cells and is mainly composed of the fibrous proteins collagen and elastin. Due to their arrangement and content, a papillary and a reticular zone are distinguished. Adjacent to the dermal–epidermal junction is the papillary dermis. The reticular dermis lies below it and forms the deeper part of the dermis. Small unstructured collagen fibrils are found within the papillary dermis, while the reticular dermis contains larger fibrils that form fiber bundles. The papillary dermis consists of finely woven collagen type III fibers, whereas the reticular dermis is dominated by fiber bundles of collagen type I (Smith et al. 1982; Guimberteau et al. 2017; Meigel et al. 1977; Weber et al. 1984). The mean cross-sectional dimensions of collagen fibers thereby increase from papillary to reticular dermis, though corresponding values vary significantly in the literature. Reported cross-sectional dimensions of fibers range from 3 to 47 $\upmu$m in the papillary dermis and from 40 to 136 $\upmu$m in the reticular dermis (Brown 1973; Ueda et al. 2019; Stewart 1995; Junqueira et al. 1983). In terms of collagen content, there is no sharp transition between the two layers, but rather a continuous increase in collagen content from the dermal–epidermal junction toward the reticular dermis. This increase can be observed further down than just until the end of the papillary layer (Wang et al. 2015). Similarly, the arrangement of collagen fibers differs with the mean in-plane orientation of collagen fibers being more pronounced the deeper they lie in the dermis (Lovell et al. 1987). Difference in composition is not restricted to collagen fibers. Depth-dependent changes in thickness (Ueda et al. 2019), orientation (Smith et al. 1982) as well as content (Wang et al. 2015) have been reported for elastin fibers, with changes similar as described for collagen. Finally, between papillary and reticular dermis there is a clear difference in fibroblast physiology, extracellular matrix production and organization as well as participation in inflammatory responses (Smith et al. 1982; Sorrell and Caplan 2004; Harper and Grove 1979).

The deepest layer of skin is the hypodermis. An abrupt transition from the fibrous dermal matrix to adipocytes-rich tissue defines the hypodermis. In fact, the hypodermis consists mainly of adipocytes supported by the interlobular septa, a collagen-based structure (Sheldon 2011; Comley and Fleck 2010).

### Multilayer model of skin

Considering its histological composition, we model skin as a layered biphasic material. With the focus on the dermal–epidermal layers, we thereby distinguish epidermis (Epi), papillary (PD) and reticular dermis (RD) as well as hypodermis (Hypo). Thickness values for the epidermis and the papillary dermis are chosen to be 100 $\upmu$m and 200 $\upmu$m, respectively (Mogensen et al. 2008; Reed and Ackerman 1973; Smith et al. 1982). The thickness of the reticular dermis is not fixed a priori but left unspecified as a parameter to evaluate the influence of different skin thicknesses. The thickness of the hypodermis was chosen to be 3 mm.

All four layers are modeled as biphasic materials according to the theory of porous media (Ehlers 2002; Ehlers et al. 2009; Stracuzzi et al. 2018). Assuming the mapping $\varvec{x} = \varvec{\chi }_\alpha (\varvec{X}_\alpha ,t)$ between material $\varvec{X}_\alpha$ and spatial $\varvec{x}$ position of a material particle of phase $\alpha$, the deformation of the solid phase is characterized by the deformation gradient $\varvec{F} = \frac{\partial \varvec{\chi }_s(\varvec{X}_s,t)}{\partial \varvec{X}_s}$. The left Cauchy–Green tensor follows as $\varvec{b} = \varvec{FF}^T$ and the volume ratio $J = \text {det}(\varvec{F})$. The constitutive equation for the solid phase of epidermis, papillary and reticular dermis is defined based on the formulation proposed by Wahlsten et al. (2019). Thus, for each layer a Rubin and Bodner-type strain energy function $\varPsi _s$ is applied to describe the mechanical contribution of the solid phase to the free energy:

1 $$\begin{aligned} \varPsi _s = \varphi _s^{\mathrm {ref}} \frac{\mu _0}{2q} \left( \exp (qg) - 1 \right) \text { with } g = g_m + g_{fe} + g_{fd} \end{aligned}$$

where $g_m, g_{fe}$ and $g_{fd}$ correspond to matrix, elastic fiber response and dissipative fiber response. They are defined as

2 $$\begin{aligned} g_m= m_1 \left[ tr(\varvec{Fb}_0\varvec{F}^T) - 3\right] + \frac{m_1}{m_2}\left[ \left( JJ_0\right) ^{-2m_2} - 1 \right] \end{aligned}$$

3 $$\begin{aligned} g_{fe}&= \frac{m_{fe}}{m_{4e}} \frac{1}{N} \sum _{i=1}^{N}\langle \lambda _{fe}^{i} - 1 \rangle ^{2m_{4e}} \end{aligned}$$

4 $$\begin{aligned} g_{fd}&= \frac{m_{fd}}{m_{4d}} \frac{1}{N} \sum _{i=1}^{N}\langle \lambda _{fd}^{i} - 1 \rangle ^{2m_{4d}}. \end{aligned}$$

where $\mu _0, q, m_1, m_2, m_{fe}, m_{4e}, m_{fd}$ and $m_{4d}$ are material parameters, $\varphi _s^{\mathrm {ref}}$ is the solid volume fraction in the reference configuration, *N* accounts for the number of fibers and $\lambda _{fe}^{i}$ and $\lambda _{fd}^{i}$ are fiber stretches for the elastic and dissipative contributions. The Macaulay brackets $\langle \cdot \rangle$ ensure fibers being only active in tension. The initial deformation state $\varvec{b}_0$ and $J_0$ therein result from initial swelling from the zero-energy state to the reference configuration, due to a nonzero osmotic pressure within each layer. Since the geometry is prescribed in the swollen state, different swelling stretches in different layers do not induce bending (Lucantonio et al. 2014).

The motion of the fluid depends on its chemical potential $\mu _F$:

5 $$\begin{aligned} \mu _F = p - \varDelta \pi \end{aligned}$$

where *p* is the fluid hydrostatic pressure and $\varDelta \pi$ the osmotic pressure. Following the approach by Ehret et al. (2017) and Stracuzzi et al. (2018), we describe the osmotic contribution constitutively as a volumetric term, which arises due to fixed charges in the tissue:

6 $$\begin{aligned} \varDelta \pi = - \frac{\partial \varPsi _{OSM}(J)}{\partial J} = \beta _0 \left( \frac{1 - \varphi _s^{\mathrm {ref}}}{J - \varphi _s^{\mathrm {ref}}}\right) ^{\beta _1}. \end{aligned}$$

Herein, $\beta _0$ and $\beta _1$ are experimentally derived material specific parameters.

Finally, fluid flow $\varvec{q}$ and fluid chemical potential $\mu _F$ are connected via Darcy’s law under the assumption of a spatially isotropic permeability tensor $\varvec{k} = k(J) \varvec{I}$.

7 $$\begin{aligned} \varvec{q}&= -\varvec{k} \text { grad}\left[ \mu _F\right] \end{aligned}$$

8 $$\begin{aligned} k(J)&= k_0 \left( \frac{J - \varphi _s^{\mathrm {ref}}}{1 - \varphi _s^{\mathrm {ref}}}\right) ^\kappa \end{aligned}$$

Further details on the model formulation and its implementation for numerical calculations are provided in Wahlsten et al. (2019) and Ehlers et al. (2009).

The formulation of the constitutive model applied to different skin layers is based on the microstructure of the papillary and reticular dermis. For these layers, a clear correspondence between the fibrous components of the model and the collagen fibers as well as the matrix and the ground substance exists. A similar direct correlation does not exist for the other two layers, i.e., epidermis and adipose tissue. Yet, the histology of these layers and the results of mechanical tests (Sommer et al. 2013) suggest that they are biphasic, dissipative and nonlinear. In addition, both layers include fibrous elements: the protein filaments present in the cytoskeleton of keratinocytes in the epidermis and the collagen structure of the hypodermis. Thus, the chosen model formulation can provide a phenomenological representation of the mechanical response of all layers.

## Experimental observations

The analysis considers a set of previously performed tensile (Wahlsten et al. 2019) and suction experiments (Pensalfini et al. 2018) (3.1). The data are complemented with in situ visualization of skin deformation during in vivo suction (3.2) and out-of-plane deflection during ex vivo uniaxial tension (3.3).

### Existing data

The multilayered model is used to rationalize ex vivo (uniaxial tension Wahlsten et al. 2019) and in vivo (suction Pensalfini et al. 2018) experimental data. Ex vivo tensile experiments on human abdominal and breast skin samples were performed in physiological saline solution (0.15 M NaCl). Specimen gauge dimensions were $20\times 5\times 2$ mm$^3$. The nominal strain rate was $0.001\text { s}^{-1}$ for monotonic tensile tests. In relaxation experiments, samples were elongated at a nominal strain rate of $0.05\text { s}^{-1}$ until a pre-defined peak force of 1.0 N was reached after which sample length was hold fixed. Tension–stretch measurements show a distinct J-shaped curve with significant volume reduction under uniaxial loading. Important for the present work, out-of-plane bending of the top surface of the skin was consistently observed in uniaxial tension experiments.

**Fig. 2:**
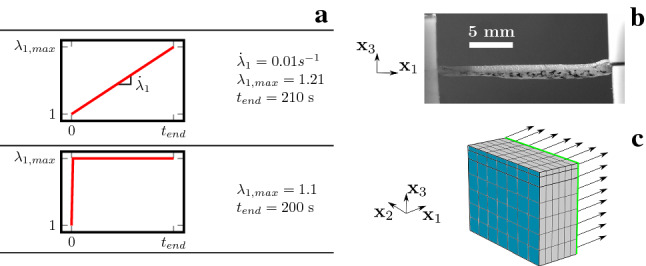
Uniaxial monotonic and relaxation protocols. **a** Loading protocol applied. **b** Illustration of the experiment (side view). **c** FE model: the mesh is depicted and the displacement boundary conditions (arrows on green face). Symmetry conditions were applied on the facing plane (blue), All other faces are traction-free

A finite element model in COMSOL Multiphysics ®(COMSOL Multiphysics$^{\textregistered }$ 5.4a, COMSOL AB, Stockholm, Sweden) using a rectangular cuboid domain of $1\times 2.5\times 2\ \text {mm}^3$ was used to simulate the uniaxial experiments, see Fig. 2. The volume was discretized with 252 hexahedral elements. Displacement and fluid chemical potential were interpolated using quadratic and linear functions, respectively. The right lateral and bottom sides were modeled with homogeneous Dirichlet boundary condition for the fluid chemical potential, while the top surface was modeled as impermeable, in line with the barrier function of the stratum corneum. Impermeability was also assumed for the longitudinal side due to the dimensions of the specimen. Symmetry condition applies for the left lateral side. As shown in Fig. 2, for the face at $x_1=0$, the x-component of the displacement was constrained while at the opposite side ($x_1=1$ mm) a x-displacement was imposed in accordance with the uniaxial monotonic and relaxation experiments. Symmetry condition was again assumed for the left lateral side, and the left bottom node was fixed to suppress any rigid body motion. All other displacement degrees of freedom of the nodes on the external faces were free, in order to represent the traction free boundary condition.

**Fig. 3:**
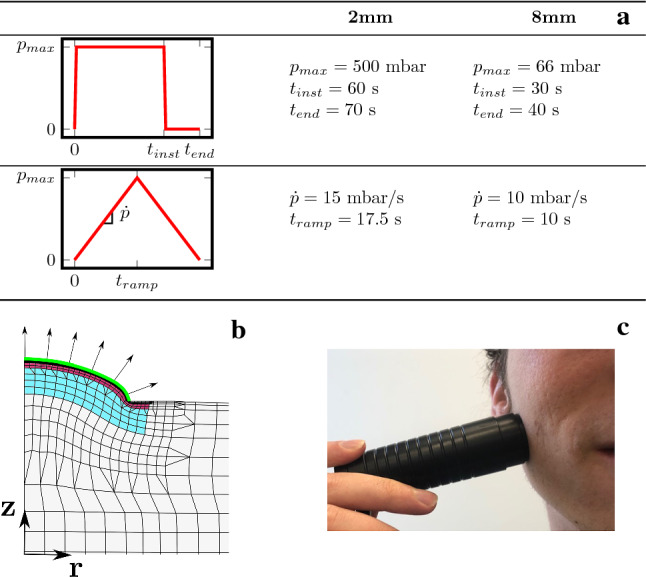
Information on suction experiments and corresponding simulation: **a** the protocols for instantaneous and linear pressure loading for 2 mm and 8 mm openings; **b** a resulting deformation of the axisymmetric simulation for the 8 mm opening indicating the epidermis (black), papillary dermis (red) and reticular dermis (blue) as well as the applied pressure (green and arrows) and **c** a picture of the Cutometer as employed during the experiments in-vivo

**Fig. 4:**
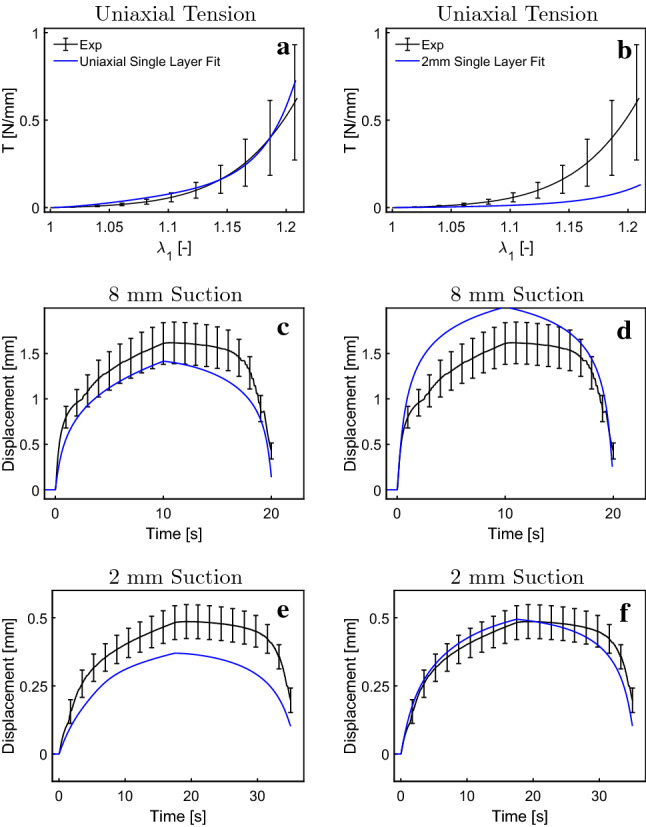
A single-layer model fitted to uniaxial tensile data (Wahlsten et al. 2019) (**a**), results in too stiff responses for 8 mm suction (**c**) and 2 mm suction (**e**); a single-layer model fitted to 2 mm suction (**f**) on the other hand results in too soft responses for 8 mm suction (**d**) and uniaxial tension (**b**)

As reported in Pensalfini et al. (2018), in vivo suction experiments on facial skin were performed using the CUTOMETER ®(Courage & Khazaka electronic GmbH, Köln, Germany). Instantaneous as well as linear pressure changes were applied using probe opening diameters of 2 mm and 8 mm. A description of the loading schemes is provided in Fig. 3. Measured skin responses as mean apex displacement–time curves are reported in Fig. 4. Linear loading shows an initial softer response which stiffens as pressure increases and a distinct remaining displacement at the end of the experiment. The latter is also observed for the instantaneous case. Additionally, we see that during the constant pressure phase in loading and unloading the apex elevation of skin shows a characteristic creep-like behavior.

The suction experiments were simulated using a 2D axisymmetric model in COMSOL Multiphysics. As shown in Fig. 3, the overall dimensions of the model for the 8 mm suction opening were $10\times 16 \ \text {mm}^2$, as required to avoid an influence of the far field boundary conditions on the simulated suction response (Nava 2007). In line with facial skin thickness measurements (Chopra et al. 2015), simulations were carried out with a thickness of 0.1 mm for epidermis, 0.2 mm for papillary dermis and 1.1 mm for reticular dermis. For the 2 mm probe, the model included a layer of hypodermis below the skin, while the 8 mm probe simulation additionally had a 2 mm layer of stiff muscle tissue at the bottom, see Fig. 3. The top surface was modeled as impermeable, while all other edges had a homogeneous Dirichlet boundary condition for the fluid chemical potential. According to previous investigations (Weickenmeier et al. 2015), frictionless contact was assumed between skin and Cutometer. The contact was assumed to be closed at all times of the simulation. The load was applied as a negative pressure. All other boundaries were assumed traction free. Part of the FE model is depicted in Fig. 3c. The top layers as well as the edge of the Cutometer require a finer mesh to avoid mesh dependence of simulation results. As for the uniaxial simulations, displacement and fluid chemical potential were interpolated using quadratic and linear functions, respectively.

As a first step, the data from uniaxial and suction experiments were analyzed using the biphasic single-layer model proposed by Wahlsten et al. (2019). Based on an optimization procedure (using MATLABs Global Optimization Toolbox MATLAB Optimization Toolbox 2018), several model parameter sets were obtained providing a good representation of the uniaxial data, as shown for a selected representative parameter set in Fig. 4. Application of all these parameter sets to simulate suction experiments provided a too stiff response for both the 8 mm and 2 mm probe, see Fig. 4a, c and e. When improving the suction response by softening the fiber stiffness in the whole material, the predicted response in uniaxial experiments is far from the measurements (data not shown). Correspondingly, a single-layer model fitted to 2-mm suction experiments results in a much too soft prediction for the monotonic uniaxial experiments and the 8-mm suction experiment, see Fig. 4b, d and f. These results indicate a possible limitation of a single-layer model in its ability to represent the results of different experiments. However, we cannot exclude that the observed discrepancies could be reduced through a global fitting procedure that aims at simultaneously optimizing the response for all experiments. This global inverse analysis was not performed here due to the large computational cost associated with all simulations. Based on this, we hypothesized that the observed contradictions may be resolved by considering the multilayered structure of the skin.

### MRI imaging of suction experiments

**Fig. 5:**
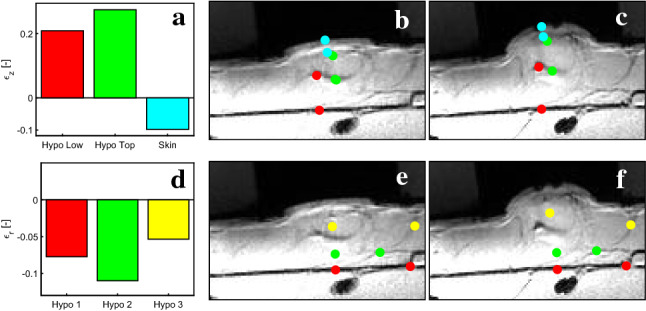
MR Imaging of suction experiments with a 10 mm probe opening. Strains in hypodermis and skin in **a** out-of-plane and **d** in-plane direction. The corresponding features tracked in the initial (**b**, **e**) and deformed configuration (**c**, **f**) show a reduction in skin’s thickness (blue in **a**) and contractile in-plane strains and thickness increase in hypodermis

In-depth visualization of deformation in suction experiments was performed using a 3 T Philips Achieva system (Philips Healthcare, Best, the Netherlands). Measurements were performed on the volar forearm of a 29-year-old female volunteer. Experiments were performed after receiving written informed consent and according to the ethics and institutional guidelines (EK 2018-N-45). A suction device (Müller et al. 2018), adapted for MRI measurements, with a diameter of 10 mm and a maximum tissue elevation of 2.5 mm was used. An image resolution of 200 $\upmu$m resulted in an MR image acquisition time of approximately 7 min. Measurements were first performed at zero suction pressure and then at the elevated state. For the latter, it was ensured that the skin was always at the maximum elevation for the whole duration required for image acquisition. Tracking of natural features allows estimating strains in out-of-plane and in-plane direction, see Fig. 5. Strains were defined as the ratio of the distance between the respective points in deformed and undeformed configuration. The hypodermis experiences large positive out-of-plane strains, which increase closer to the reticular dermis. Experiments show a decrease in skin thickness of about 10%. In-plane strains at different depths of the hypodermis are all negative, confirming that the tissue is pulled toward the center of the suction region.

### Quantification of out-of plane deformation in tensile experiments

**Fig. 6:**
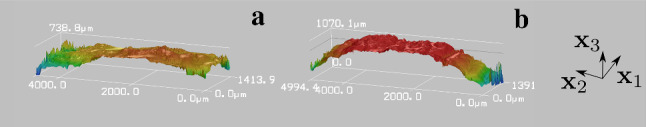
Skin shows an out-of-plane deflection under uniaxial tension: unstretched state already showing an out-of-plane curvature (**a**), which considerably increases when stretched uniaxially in $x_1$ direction (**b**)

Based on the hypothesis that out-of-plane deflection during uniaxial tensile tests might be associated with the multilayered structure of the skin, measurements were preformed to quantify this deformation. Experiments were conducted using a 3D Laser Scanning Confocal Microscope (Keyence Cooperation, Osaka, Japan). Human skin biopsies were accessed through the Dermatology department of the University Hospital Zurich with assistance of the SKINTEGRITY biobank. The donors provided signed informed consent that was approved by the local institutional review board (EK 647 and EK 800). The use of surplus skin for biomechanical experiments had been approved by the Ethical Committee of Canton Zurich (BASEC ID: 2017-00684). Skin biopsies were cut into specimens of $40\times 5$ mm$^2$. The thickness of the skin samples was approximately 2 mm. Experiments were performed with specimens partially submerged in a physiological saline solution, with the epidermal surface emerging out of the liquid to enable optical out-of-plane deflection measurements. The skin sample was elongated from an initially untaut state with increments of 1.25% nominal strain. Each acquisition required approximately 5 min. Measurements confirmed expectations: the skin shows an out-of-plane curvature in the unloaded state already and the out-of-plane deflection increases as extension is applied, as shown in Fig. 6. For the deflection, the difference between the out-pf-plane deformation of the center and points 2 mm from the center was taken.

## Results of multilayer model simulations

### The multilayer model describes uniaxial and suction experiments

**Fig. 7:**
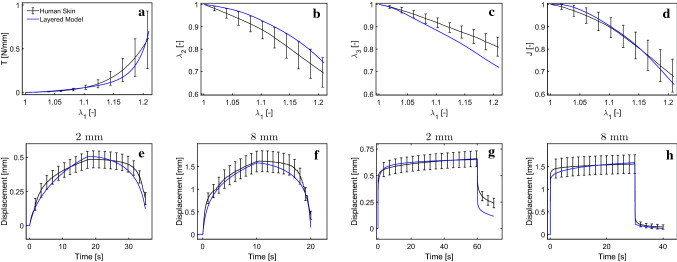
Uniaxial tensile (**a**–**d**), 2 mm suction (**e**, **f**) and 8 mm suction (**g**, **h**) response of human skin. The model well represents the J-shaped stress–strain curve (**a**), the large lateral (**b**) and vertical (**c**) contraction, as well as linear (**e**, **f**) and instantaneous (**g**, **h**) suction responses for both probe opening diameters

**Fig. 8:**
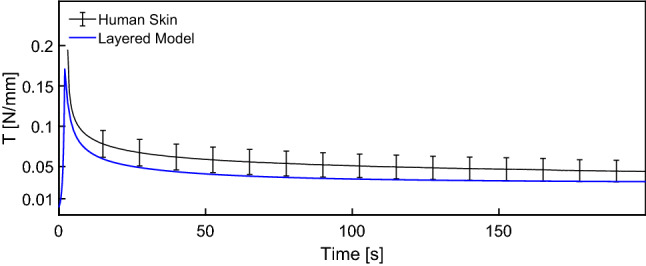
Model reproduces the observed uniaxial relaxation behavior of human skin

**Fig. 9:**
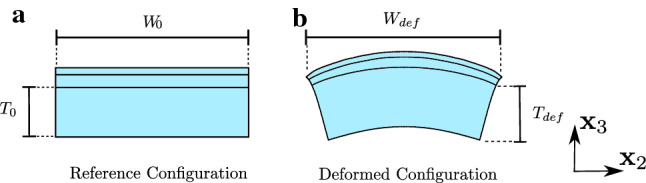
Extraction of apparent global lateral stretch $\lambda _2$ ($\lambda _2 =\frac{W_{def}}{W_0}$) and vertical stretch $\lambda _3$ ($\lambda _3=\frac{T_{def}}{T_0}$) from simulations results, for comparison with experimental data

Model parameters for each layer were determined based on an iterative procedure aiming at representing ex vivo uniaxial as well as in vivo suction observations. The corresponding parameter values are reported in Table 1. The multilayered model leads to a good agreement for all measured curves, as shown in Fig. 7. The distinct J-shaped tension–stretch relationship as well as the volume loss upon uniaxial stretching is well reproduced. Note that due to the newly resulting out-of-plane deflection in the simulations, comparison with experimental data used the following definition for the apparent global contraction stretches $\lambda _2^a$ and $\lambda _3^a$: $\lambda _2^a = \frac{W_{def}}{W_0}$ and $\lambda _3^a = \frac{T_{def}}{T_0}$, with the corresponding lengths defined in Fig. 9. For $\lambda _3^a$ only the lower part of the reticular dermis was considered to be consistent with experiments. Strong stress reduction within the first two minutes during relaxation experiments is well captured as depicted in Fig. 8.

The new model also well describes the experimental results for linear and instantaneous suction experiments for 2 mm and 8 mm probe opening diameters, as shown in Fig. 7. Especially for the 8 mm probe, it captures the full loading path very well. For the linear loading, the apex displacement is under-predicted during the unloading phase and the apex height drops faster after the peak compared to the experimental results. For the instantaneous loading, the apex displacement drop at the end is well captured by the 8 mm simulation, whereas the 2 mm simulation overestimates it. In the suction experiments, the highest strains occur at the surface of the skin. Thus, the upper layers, i.e., papillary dermis and epidermis, strongly influence the results of these experiments. The fluid permeability of these two layers proved to be an important parameter in modeling the viscous behavior during the instantaneous response as well as the remaining apex displacement at the end of the simulation for both experiments. This behavior is more pronounced for the 2 mm than for the 8 mm probe opening.

### Layer-specific material parameters

**Table 1:** Material parameters for each layer of the biphasic model used for representing uniaxial and suction experiments

	Epidermis	Papillary dermis	Reticular dermis	Hypodermis	Muscle tissue
$\mu _0 \text { }(\text {MPa})$	$0.508\times 10^{-2}$	$2.0608\times 10^{-2}$	$0.668\times 10^{-2}$	$2\times 10^{-4}$	$10.668 \times 10^{-2}$
*q*	3.4	3.4	3.4	1.4	3.4
$m_1$	0.2	0.1	0.23	0.05	0.1725
$m_2$	2	1	1	0.1	1
$m_{fe}$	25	110	240	50	0
$m_{fd}$	120	250	1150	50	0
$m_{4e}$	2.5	1.415	1.34	1.4	0
$m_{4d}$	1.34	1.34	1.34	1.4	0
$\vartheta \text { } (^{\circ })$	30	10	7	10	0
$k_{fd} \text { }(\text {mm}^2 \text {N}^{-1} \text {s}^{-1})$	$1.43\times 10^{-2}$	$1.43\times 10^{-1}$	$1.43\times 10^{-2}$	5.43	0
$k_0\text { } (\text {mm}^4 \text {N}^{-1} \text {s}^{-1})$	0.005	0.05	5	15	0.5
$\kappa$	2	2	2	2	2
$\beta _0 \text { }(\text {MPa})$	$2.19\times 10^{-3}$	$2.49\times 10^{-3}$	$2.49\times 10^{-3}$	$2.49\times 10^{-4}$	$2.49\times 10^{-3}$
$\beta _1$	2	2	2	2	2
$\varphi _S^{ref}$	0.3	0.3	0.3	0.3	0.3

The parameters refer to the model formulation in Eqs. (1)–(8) and Wahlsten et al. (2019)

The results of the parameter fit are reported in Table 1. These result in a stiff reticular dermis, a softer papillary dermis and a very soft epidermis. This difference is mainly reflected in the elastic fiber stiffness $m_{fe}$. The matrix properties were generally similar for all three layers. The permeability of the interstitial fluid reduces continuously from reticular to papillary dermis to epidermis. The out-of-plane orientation of the fibers on the other hand increases from reticular dermis to epidermis. While in the reticular dermis fibers have an out-of-plane angle of only 8$^\circ$, resulting in a strongly anisotropic material, the out-of-plane angle of 30$^\circ$ in the epidermis produces an almost isotropic response.

Hypodermis and muscle tissue are modeled using the same model formulation. Neither of the two layers has a significant influence on the mechanical response in the experiments considered. Thus, the simulations do not allow to evaluate corresponding model parameters for these tissues. Hence, an order of magnitude estimation of the parameters was performed using information available in the literature (Weickenmeier et al. 2015) as well as data from own experiments.

### Layer-specific analysis of uniaxial tension experiments

**Fig. 10:**
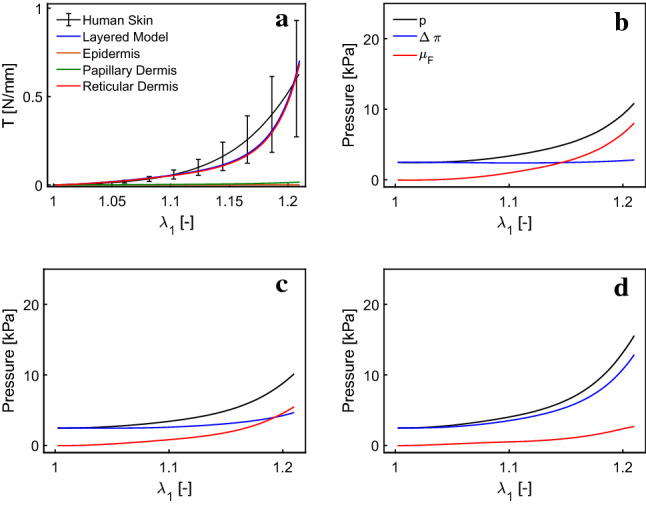
Multilayer model provides the tension–stretch curve for each layer (**a**), and predicts a heterogeneous distribution of $\varDelta \pi$, $\mu _F$ and *p* in epidermis (**b**), papillary dermis (**c**), reticular dermis (**d**), during monotonic uniaxial tensile test

To investigate the layer-specific response in tension, we calculated the nominal tension (force per unit width) for each layer during a uniaxial tension simulation. Figure 10a shows the tension distribution in the layers of skin for the monotonic uniaxial test. All three layers show a J-shaped tension–stretch response with the stiffness increasing from epidermis to papillary dermis to reticular dermis. The dominant role of the reticular dermis is evident bearing almost the entire load.

The multilayer model predicts a heterogeneous distribution of osmotic pressure, fluid chemical potential and hydrostatic pressure during uniaxial experiments, as shown in Fig. 10. Quantities are evaluated in the center of the respective layer. Osmotic pressure increases from epidermis to reticular dermis as a consequence of the out-of-plane deflection and the thereby induced non-homogeneous deformation. The stiff collagen fibers induce a larger volume change in the reticular dermis, which results from loss of interstitial fluid and therefore an increase in osmotic pressure. The fluid chemical potential decreases from the epidermis to the deeper layers, thus driving fluid flux from epidermis down toward the reticular dermis.

**Fig. 11:**
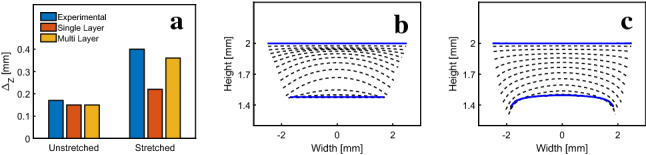
Layered model predicts the out-of-plane deflection for an initially curved skin sample (ö) and also shows a deflected final configuration for a flat initial condition (**c**) in contrast with the single-layer model (**b**)

The uniaxial experiments presented in Fig. 6 show an increase in ouf-of-plane bending of the skin under uniaxial tension. The multilayer model predicts this increase in contrast with a single-layer model as shown in Fig. 11a. When starting the simulations from a flat surface, the single-layer model initially predicts out-of-plane bending from a downwards transient fluid flux (due to the impermeable condition on the epidermal surface). In equilibrium, however, the single-layer model returns to the flat state, as shown in 11b. The multilayer model, shown in (c), in contrast, captures the observed out-of-plane bending in equilibrium. Out-of-plane deflection depends on the relative thickness of the upper layers and the reticular dermis. Corresponding simulations showed that a thinner reticular dermis results in increased and a thicker reticular dermis in a decreased out-of-plane deflection (not shown).

### Multilayer analysis of suction experiments

**Fig. 12:**
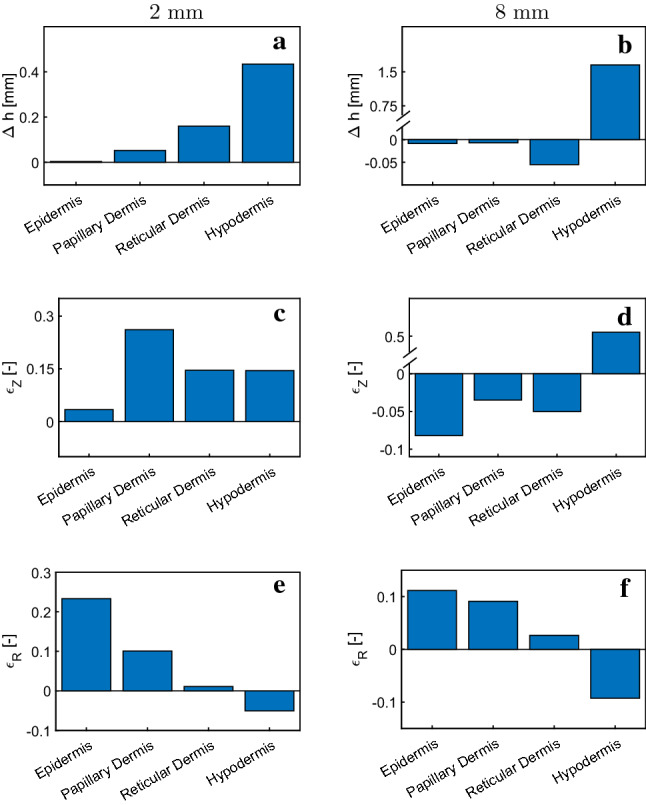
Change in thickness for different layers in **a** 2 mm and **b** 8 mm instantaneous suction simulation as well as average strains in vertical (**c**, **d**) and in-plane (radial) direction (**e**, **f**)

The multilayer model allows to quantify layer-specific deformations in suction experiments, shown in Fig. 12. Change of thickness and strains are evaluated on the symmetry axis of the simulated domain. The simulation highlights important differences between suction with a small and a large probe. For small probe openings, papillary and reticular dermis show an increase in thickness. The largest out-of-plane strain occurs in the papillary dermis, underlying its importance for this deformation state. For large probe openings, the thickness of all three skin layers decreases. Fibers are stretched due to an in-plane biaxial stress state, resulting in a contraction in vertical direction. Hypodermis moves significantly in out-of-plane direction. Yet, it imposes almost no resistance to the deformation due to its high compliance in comparison with the upper layers. The computational results are in line with the experimental observation presented in Fig. 5. Both simulations and experimental observations show a reduction in skin thickness during suction for large probe openings. With an observed reduction of approximately 10 % in experiments, numerical results are well in line with the observations.

**Fig. 13:**
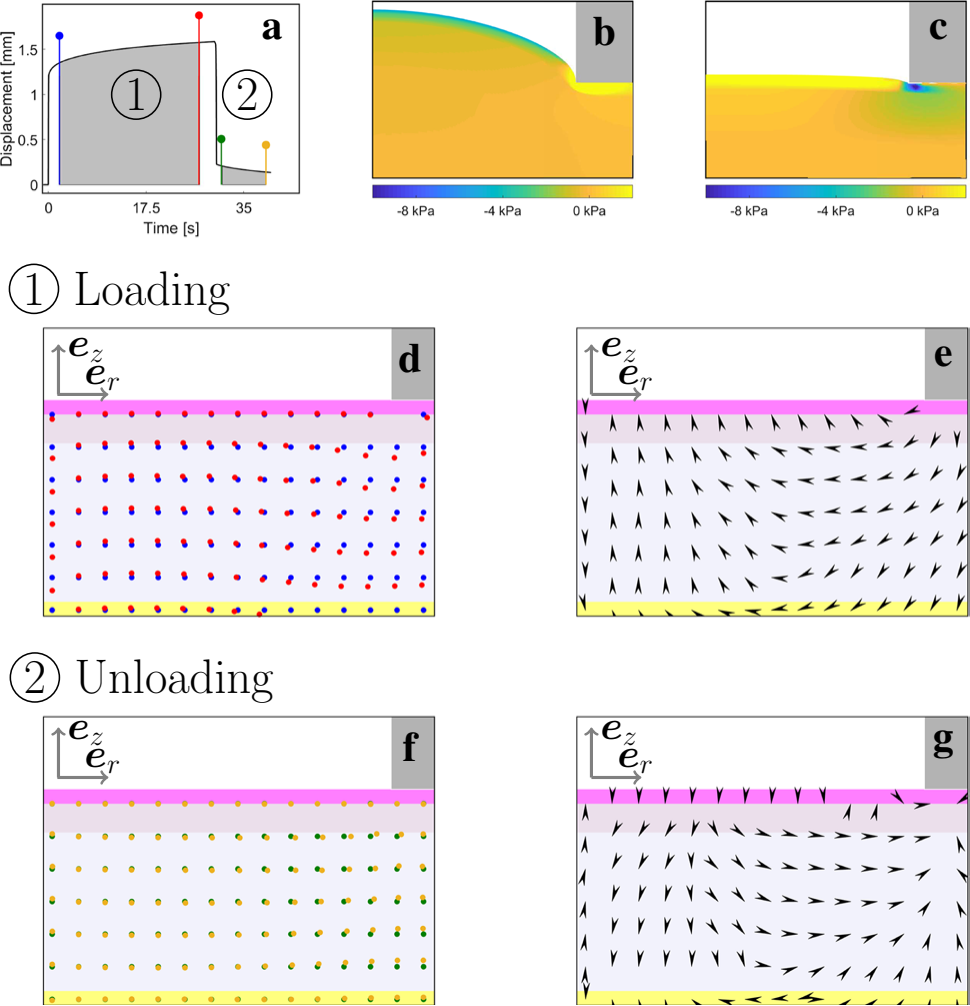
Tracing of fluid particles shows the importance of fluid flow on the time-dependent response in suction experiments. **a** Instantaneous suction response with time periods of particle tracing. Fluid chemical potential for **b** 17.5 s and **c** 35 s induces fluid flow. Start and end position of fluid particles and the derived flow field in the material frame for the loading (**d**, **e**) and the unloading period (**f**, **g**)

The creep and relaxation of the skin observed in suction experiments depends on the motion of interstitial fluid. Gradients in the fluid chemical potential within the tissue are the driving force for fluid flux and influence the observed creep as well as relaxation behavior, as shown in Fig. 13b, c. Tracing of fluid particles during corresponding simulations enables the visualization of this effect. Fluid motion in the spatial domain results from motion with the solid and interstitial flux; thus, the new position $\mathbf {x}_f$ of a fluid particle after timestep $\varDelta t$ is given by $\mathbf {x}_f(t + \varDelta t) = \mathbf {x}_f(t) + \mathbf {v}_s \varDelta t + \frac{1}{\varphi _f}\mathbf {q} \varDelta t$, with $\mathbf {v}_s$ and $\mathbf {q}$ being the spatial velocity field of the solid particles and the spatial velocity field of the fluid particles, respectively, and $\varphi _f$ the current fluid volume fraction. Fluid motion is visualized in the undeformed geometry (material description) for the phases of constant maximum pressure (d) and during zero pressure (e). Corresponding flow fields derived from fluid displacements are shown in (f) and (g) and underline the overall fluid motion from the far field toward the center during constant maximum pressure and vice versa during zero pressure. Integrating fluid particle velocities over one full cycle reveals residual fluid displacement after unloading, associated with residual deformation. Interestingly, in both limiting cases of zero and infinite permeability the apex displacement vanishes at the end of one cycle, minimizing final fluid displacement (data not shown). In fact, if the permeability is zero, no flux is possible, while the opposite case results in fast flow toward the center during loading, but also fast outflow from the center during unloading, resulting in complete recovery of apex displacement.

## Discussion

### Layer-specific mechanical behavior

Linearized stiffness values for human skin in the literature vary significantly, from few kPa to several MPa depending on the chosen deformation state and the applied experimental method. For in vivo indentation, stiffness values range from 1 to 10 kPa (Pailler-Mattei et al. 2008; Bader and Bowker 1983; Delalleau et al. 2006). Small-strain shear values for skin fall within this order of magnitude, with reported values of 0.5 kPa (Bader and Bowker 1983) and 2 kPa (Weickenmeier et al. 2015). Much higher values of stiffness are found for in vivo suction and ex vivo tensile tests, ranging from several hundred kPa in suction (Diridollou et al. 2000) to several MPa in ex vivo uniaxial tensile tests (Ní Annaidh et al. 2012; Ottenio et al. 2015). To compare our results, we linearize the quasi-static uniaxial stress–strain responses ($\dot{\epsilon } = 0.001$ s$^{-1}$) of each individual layer at small, intermediate and large strains with $\lambda _s \approx 1$, $\lambda _i \approx 1.10$ and $\lambda _l \approx 1.20$, respectively. Tangent stiffness is then calculated from nominal Tension *T* with $E = \frac{1}{t}\frac{\partial T}{\partial \lambda }$ and *t* being the initial thickness of the sample. The reticular dermis, the stiffest layer, exhibits $E_{RD,s} = 170$ kPa, $E_{RD,i} = 588$ kPa and $E_{RD,l} = 11$ MPa. Corresponding values for papillary dermis are $E_{PD,s} = 93$ kPa, $E_{PD,i} = 200$ kPa and $E_{PD,l} = 1.3$ MPa and for epidermis $E_{Epi,s} = 14$ kPa, $E_{Epi,m} = 35$ kPa and $E_{Epi,l} = 77$ kPa. Linearized tangent stiffness values thus confirm expectations from the well-known J-shaped stress–strain curve for soft biological tissues, and that our values fall within the wide scatter of previously reported data. Only few layer-specific values are found in literature. Hendriks et al. (2006) used suction to distinguish between the reticular dermis and the upper layers and fitted a neo-Hookean model to each layer. They report a 1000 times higher stiffness of reticular dermis and thus a larger difference for small strains than our model. The neo-Hookean model, however, does not offer the ability to represent volume changes or to distinguish between differences in layer-specific stiffening behavior for large strains. Thus, applying a neo-Hookean model for large strains might result in an overestimation of stiffness differences for small strains. Instantaneous Young’s moduli calculated from indentation and a fit to an Ogden model by Crichton et al. (2011) resulted in a tenfold stiffer dermis than epidermis. Moreover, a larger value of the exponent of the dermis in Crichton et al. (2011) results in a stronger stiffening behavior for large strains and thus supports our prediction that differences in linearized tangent stiffness between the layers increase with applied strain. Numerical studies on skin wrinkling often differentiate between papillary and reticular dermis (Zhao et al. 2020; Kuwazuru et al. 2008). The reported Young’s moduli of the layers differ by a factor of two. Interestingly, in contrast with our model, they often assume an even larger Young’s modulus for the epidermis. Ex vivo shear experiments (Soetens et al. 2018; Lamers et al. 2013) indicate a higher value of shear stiffness in the papillary dermis than in the reticular dermis. These findings are in line with the data reported in Ventre et al. (2009) based on ex vivo oscillatory shear experiments. For small loading magnitude, these results are captured by our model with shear strains being larger in reticular dermis than in papillary dermis (data not shown).

As indicated in Sect. 2.2 and motivated by the layers’ distinct microstructure, different model formulations could have been selected for different layers. However, using the same strain energy function allows to directly relate differences in model parameters with histological evidence. In fact, the cellular structure of the epidermis is expected to result in a less pronounced anisotropy and nonlinearity of the stress–strain curves, when compared to the dermal layers. Indeed, the corresponding out-of-plane angle of the fiber component of the model is larger, and fiber’s stiffness and nonlinearity are lower with respect to papillary and reticular dermis (see Table 1). Moreover, fluid motion is likely to be hindered more in the epidermis, which is reflected by a lower permeability. Similar conclusions can be drawn for the adipose tissue, which displays weak anisotropy and low stiffness, associated with softer fibers with respect to their matrix.

### Layer-specific properties at physiological deformation levels

**Fig. 14:**
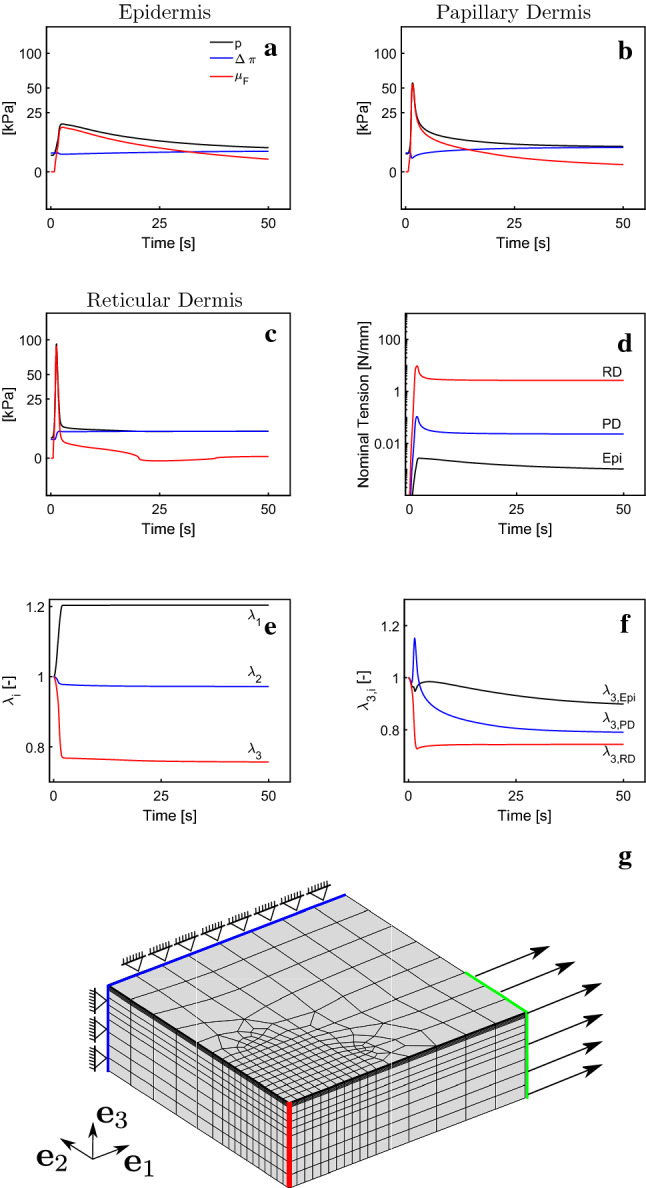
Simulation of physiological deformation provides layer-specific results. Fluid chemical potential ($\mu _F$) shows highest peak in the reticular dermis (**c**) and lowest in the epidermis (**a**) with values for papillary dermis lying in between (**b**); reticular dermis also exhibits the largest long-term changes in osmotic ($\varDelta \pi$) and hydrostatic (*p*) pressure (**c**); stretch in direction $\mathbf {e}_1$ induces small skin in-plane and large out-of-plane contractions (**e**); highest levels of vertical contraction occur in the reticular dermis (**f**); tension in the reticular dermis is several orders of magnitude larger than in the other two layers (**d**); the simulation domain ($\frac{1}{4}$ with corresponding symmetry conditions) is shown in (**g**) with applied load (green), lateral boundary conditions (blue). The pressures are evaluated on the red line in the center point of each layer; Note the nonlinear scale in (**a**)–(**c**)

The new model allows analyzing layer-specific changes associated with physiological levels of skin deformation. To this end we simulated an in vivo condition under physiological stretch as it occurs, for example, during bending and stretching of the volar forearm (Maiti et al. 2016). We considered epidermis, papillary and reticular dermis as well as hypodermis. Hypodermis was free to slide in the in-plane directions, but constrained in out-of-plane direction. To prevent influence of boundary effects, we modeled a large tissue portion and imposed in-plane constraints in the far field. The simulation domain is depicted in Fig. 14g. The tissue is deformed rapidly within 2 s to 20 % uniaxial strain and kept at that position for the remainder of the simulation. Interestingly, we see that the reduction in the thickness as described in similar in vivo experiments (Maiti et al. 2016) is reproduced by our model, as shown in Fig. 14e, f. Furthermore, all three layers show a fast rise in hydrostatic pressure, shown in (a), (b) and (c), indicating an instantaneous isochoric response, with the highest peak occurring in the reticular dermis. Fluid chemical potential drops quickly to low levels in the papillary and the reticular dermis. In the epidermis, however, the relaxation is much slower. Similar levels of hydrostatic pressure are encountered in all three layers at the end of the simulation, with the highest pressure occurring again in the reticular dermis. The reticular dermis is also responsible for limiting the deformation since it bears most of the load, as shown in (d).

### Model limitations

The present model includes several simplifications and limitations. First, it should be highlighted that the material parameters reported in Table 1 represent one combination of parameter values among several which might yield a prediction of similar quality. In fact, the existence of several minima for the hypersurface of the optimization cost function is typical for this type of inverse analysis (Evans and Avril 2012). Appendix reports the influence of selected model parameters on the uniaxial and suction response. A significant limitation is also the fact that the model is only calibrated using a few specific deformation modes and rates. Indentation (Virén et al. 2018; Abellan et al. 2013; Iivarinen et al. 2014) and shear (Soetens et al. 2018; Lamers et al. 2013; Geerligs et al. 2011) are relevant deformation modes not considered in the present inverse analysis. Furthermore, dissipative processes observed during fast cyclic shear loading (Lamers et al. 2013) are not fully captured by our model, which considers dissipative effects only through time-dependent fiber response and fluid flux. Note in this respect that dissipation in the original formulation of the Rubin–Bodner model (Rubin and Bodner 2002) was based on the distortional deformation of the matrix, which is relevant for shear deformations. Corresponding modifications of the present model equations could thus allow to reproduce cyclic shear observations.

A further important limitation is the fact that we neglect possible mechanical and hydraulic anisotropy. We model skin as a mechanically in-plane isotropic material. Due to preferred orientation of collagen fibers, especially in the dermis, skin is known to exhibit in-plane anisotropic behavior (Ní Annaidh et al. 2012; Ehret et al. 2012; Ridge and Wright 1966; Ueda et al. 2019). Similarly, we apply an isotropic permeability tensor. It was shown that the anisotropic organization of fibers in soft tissues leads to anisotropic hydraulic properties (Wellen et al. 2004). Formulations of anisotropic permeability tensors for large deformation exist in the literature (Ateshian and Weiss 2010; Federico and Herzog 2008; Federico and Grillo 2012) and might lead to a more accurate description of fluid motion within the tissue.

Finally, we assume sharp and flat in-plane interfaces between the skin layers. While an abrupt transition might be a realistic assumption for the interface between reticular dermis and hypodermis (Ruth and Freinkel 2001), this is not valid for the other boundaries. The change of stiffness in the dermis is likely to be more gradual between papillary and reticular layer as collagen content changes continuously along the thickness (Wang et al. 2015). In the same manner, the dermal–epidermal junction is an undulated surface, which flattens upon physiological applied strain (Maiti et al. 2016) and thus questions whether a sharp flat interface is a valid model assumption. Since flattening of the dermal–epidermal junction occurs before a stretching of epidermal cells during physiological deformations the current model might underestimate the actual stiffness of the epidermis (Maiti et al. 2016; Ferguson and Barbenel 1981).

## Conclusions

A biphasic multilayer model of skin was proposed and implemented for finite element simulations. The model rationalizes observations from ex vivo tension and in vivo suction experiments. The obtained distributions of properties indicate that: (a) the reticular dermis is the stiffest layer in tension, bearing almost all load in uniaxial tensile tests; (b) the softer papillary dermis and epidermis ensure a more compliant response in suction experiments; (c) the differences in mechanical properties of the layers are responsible for out-of-plane curvature during ex vivo uniaxial tensile experiments; (d) the low permeability in epidermis and papillary dermis results in stronger hindrance for fluid flow which determines time-dependent phenomena in suction experiments. The model further predicts layer-specific deformations and changes in osmotic and hydrostatic pressures associated with each specific loading mode, and this information is important for the analysis of mechanotransduction in human skin. Several model assumptions and simplifications were identified for which future work is required, provided corresponding experimental information.

## Supplementary Information

Below is the link to the electronic supplementary material.

Supplementary material 1 (pdf 638 KB)

## References

[CR1] Abellan MA, Zahouani H, Bergheau JM (2013). Contribution to the determination of in vivo mechanical characteristics of human skin by indentation test. Comput Math Methods Med.

[CR2] Abellan MA, Bergheau JM, Zahouani H (2014) Numerical simulation of the influence on interstitial fluid flow and ion transport of the viscous mechanical behavior of human skin In Vivo. In: 11th World congress on computational mechanics, WCCM 2014, 5th European conference on computational mechanics, ECCM 2014 and 6th European conference on computational fluid dynamics, ECFD 2014

[CR3] Ateshian GA, Weiss JA (2010). Anisotropic hydraulic permeability under finite deformation. J Biomech Eng.

[CR4] Bader DL, Bowker P (1983). Mechanical characteristics of skin and underlying tissues in vivo. Biomaterials.

[CR5] Barbarino GG, Jabareen M, Mazza E (2011). Experimental and numerical study on the mechanical behavior of the superficial layers of the face. Skin Res Technol.

[CR6] Barnes LA, Marshall CD, Leavitt T, Hu MS, Moore AL, Gonzalez JG, Longaker MT, Gurtner GC (2018) Mechanical forces in cutaneous wound healing: emerging therapies to minimize scar formation 10.1089/wound.2016.0709 PMC5792236 29392093

[CR7] Beldie L, Walker B, Lu Y, Richmond S, Middleton J (2010). Finite element modelling of maxillofacial surgery and facial expressions—a preliminary study. Int J Med Robot Comput Assist Surg.

[CR8] Benítez JM, Montáns FJ (2017). The mechanical behavior of skin: structures and models for the finite element analysis. Comput Struct.

[CR9] Bhushan B, Tang W, Ge S (2010). Nanomechanical characterization of skin and skin cream. J Microsc.

[CR10] Bischoff JE, Arruda EM, Grosh K (2000). Finite element modeling of human skin using an isotropic, nonlinear elastic constitutive model. J Biomech.

[CR11] Briggaman RA, Wheeler CE (1975). The epidermal dermal junction. J Investig Dermatol.

[CR12] Brown IA (1973). A scanning electron microscope study of the effects of uniaxial tension on human skin. Br J Dermatol.

[CR13] Buganza Tepole A, Kuhl E (2016). Computational modeling of chemo-bio-mechanical coupling: a systems-biology approach toward wound healing. Comput Methods Biomech Biomed Eng.

[CR14] Buganza Tepole A, Joseph Ploch C, Wong J, Gosain AK, Kuhl E (2011). Growing skin: a computational model for skin expansion in reconstructive surgery. J Mech Phys Solids.

[CR15] Burgeson RE, Christiano AM (1997). The dermal-epidermal junction. Curr Opin Cell Biol.

[CR16] Chen X, Li J, Li J, Li Q, Zhang W, Lei Z, Qin D, Pan Z, Li X (2019) Spatial-temporal changes of mechanical microenvironment in skin wounds during negative pressure wound therapy. ACS Biomater Sci Eng 10.1021/acsbiomaterials.8b01554 33405552

[CR17] Chopra K, Calva D, Sosin M, Tadisina KK, Banda A, De La Cruz C, Chaudhry MR, Legesse T, Drachenberg CB, Manson PN, Christy MR (2015). A comprehensive examination of topographic thickness of skin in the human face. Aesthetic Surg J.

[CR18] Comley K, Fleck NA (2010). A micromechanical model for the Young’s modulus of adipose tissue. Int J Solids Struct.

[CR19] Crichton ML, Donose BC, Chen X, Raphael AP, Huang H, Kendall MA (2011). The viscoelastic, hyperelastic and scale dependent behaviour of freshly excised individual skin layers. Biomaterials.

[CR20] Delalleau A, Josse G, Lagarde JM, Zahouani H, Bergheau JM (2006). Characterization of the mechanical properties of skin by inverse analysis combined with the indentation test. J Biomech.

[CR21] Delalleau A, Josse G, Lagarde JM, Zahouani H, Bergheau JM (2008). A nonlinear elastic behavior to identify the mechanical parameters of human skin in vivo. Skin Res Technol.

[CR22] Diridollou S, Berson M, Vabre V, Black D, Karlsson B, Auriol F, Gregoire J, Yvon C, Vaillant L, Gall Y, Patat F (1998). An in vivo method for measuring the mechanical properties of the skin using ultrasound. Ultrasound Med Biol.

[CR23] Diridollou S, Patat F, Gens F, Vaillant L, Black D, Lagarde JM, Gall Y, Berson M (2000). In vivo model of the mechanical properties of the human skin under suction. Skin Res Technol.

[CR24] Ehlers W (2002) Foundations of multiphasic and porous materials. Porous Media. Springer, Berlin Heidelberg, Berlin, Heidelberg, pp 3–86

[CR25] Ehlers W, Karajan N, Markert B (2009). An extended biphasic model for charged hydrated tissues with application to the intervertebral disc. Biomech Model Mechanobiol.

[CR26] Ehret AE, Hollenstein M, Mazza E, Itskov M (2012). Porcine dermis in uniaxial cyclic loading: sample preparation, experimental results and modeling. J Mech Mater Struct.

[CR27] Ehret AE, Bircher K, Stracuzzi A, Marina V, Zündel M, Mazza E (2017). Inverse poroelasticity as a fundamental mechanism in biomechanics and mechanobiology. Nat Commun.

[CR28] Evans ND, Oreffo RO, Healy E, Thurner PJ, Man YH (2013). Epithelial mechanobiology, skin wound healing, and the stem cell niche. J Mech Behav Biomed Mater.

[CR29] Evans S, Avril S (2012). Editorial: identification of material parameters through inverse finite element modelling. Comput Methods Biomech Biomed Eng.

[CR30] Federico S, Grillo A (2012). Elasticity and permeability of porous fibre-reinforced materials under large deformations. Mech Mater.

[CR31] Federico S, Herzog W (2008). On the permeability of fibre-reinforced porous materials. Int J Solids Struct.

[CR32] Ferguson J, Barbenel JC (1981). Skin surface patterns and the directional mechanical properties of the dermis.

[CR33] Flynn C, McCormack BAO (2008). Finite element modelling of forearm skin wrinkling. Skin Res Technol.

[CR34] Flynn C, Taberner A, Nielsen P (2011). Mechanical characterisation of in vivo human skin using a 3D force-sensitive micro-robot and finite element analysis. Biomech Model Mechanobiol.

[CR35] Geerligs M, Oomens C, Ackermans P, Baaijens F, Peters G (2011). Linear shear response of the upper skin layers. Biorheology.

[CR36] Guimberteau JC, Delage JP, Sawaya E (2017). The architectural behavior of the skin. Agache’s measuring the skin.

[CR37] Harper RA, Grove G (1979). Human skin fibroblasts derived from papillary and reticular dermis: differences in growth potential in vitro. Science (New York, NY).

[CR38] Hendriks F, Brokken D, Oomens C, Bader D, Baaijens F (2006). The relative contributions of different skin layers to the mechanical behavior of human skin in vivo using suction experiments. Med Eng Phys.

[CR39] Iivarinen JT, Korhonen RK, Jurvelin JS (2014). Experimental and numerical analysis of soft tissue stiffness measurement using manual indentation device—significance of indentation geometry and soft tissue thickness. Skin Res Technol.

[CR40] Joodaki H, Panzer MB (2018). Skin mechanical properties and modeling: a review. Proc Inst Mech Eng [H].

[CR41] Junqueira LC, Montes GS, Martins JE, Joazeiro PP (1983) Dermal collagen distribution—a histochemical and ultrastructural study. Histochemistry 79 10.1007/BF00491775 6654703

[CR42] Kuwazuru O, Saothong J, Yoshikawa N (2008). Mechanical approach to aging and wrinkling of human facial skin based on the multistage buckling theory. Med Eng Phys.

[CR43] Lamers E, van Kempen TH, Baaijens FP, Peters GW, Oomens CW (2013). Large amplitude oscillatory shear properties of human skin. J Mech Behav Biomed Mater.

[CR44] Lee T, Turin SY, Gosain AK, Tepole AB (2018). Multi-view stereo in the operating room allows prediction of healing complications in a patient-specific model of reconstructive surgery. J Biomech.

[CR45] Limbert G (2017). Mathematical and computational modelling of skin biophysics: a review. Proc R Soc A Math Phys Eng Sci.

[CR46] Lovell C, Smolenski K, Duance V, Light N, Young S, Dyson M (1987). Type I and III collagen content and fibre distribution in normal human skin during ageing. Br J Dermatol.

[CR47] Lucantonio A, Nardinocchi P, Pezzulla M (2014). Swelling-induced and controlled curving in layered gel beams. Proc R Soc A Math Phys Eng Sci.

[CR48] Maiti R, Gerhardt LC, Lee ZS, Byers RA, Woods D, Sanz-Herrera JA, Franklin SE, Lewis R, Matcher SJ, Carré MJ (2016). In vivo measurement of skin surface strain and sub-surface layer deformation induced by natural tissue stretching. J Mech Behav Biomed Mater.

[CR49] MATLAB Optimization Toolbox (2018) Matlab optimization toolbox. The MathWorks, Natick, MA, USA

[CR50] Meigel WN, Gay S, Weber L (1977). Dermal architecture and collagen type distribution. Arch Dermatol Res.

[CR51] Mogensen M, Morsy HA, Thrane L, Jemec GB (2008). Morphology and epidermal thickness of normal skin imaged by optical coherence tomography. Dermatology.

[CR52] Mollemans W, Schutyser F, Nadjmi N, Maes F, Suetens P (2007). Predicting soft tissue deformations for a maxillofacial surgery planning system: from computational strategies to a complete clinical validation. Med Image Anal.

[CR53] Montagna W, Parakkal PF (1974) The structure and function of skin. Academic Press

[CR54] Müller B, Elrod J, Pensalfini M, Hopf R, Distler O, Schiestl C, Mazza E (2018). A novel ultra-light suction device for mechanical characterization of skin. PLoS ONE.

[CR55] Nava A (2007) In vivo characterization of the mechanical response of soft human tissue. Ph.D. thesis, ETH Zurich

[CR56] Ní Annaidh A, Bruyère K, Destrade M, Gilchrist MD, Otténio M (2012). Characterization of the anisotropic mechanical properties of excised human skin. J Mech Behav Biomed Mater.

[CR57] Oftadeh R, Connizzo BK, Nia HT, Ortiz C, Grodzinsky AJ (2018). Biological connective tissues exhibit viscoelastic and poroelastic behavior at different frequency regimes: application to tendon and skin biophysics. Acta Biomater.

[CR58] Oomens C, van Campen D, Grootenboer H (1987). A mixture approach to the mechanics of skin. J Biomech.

[CR59] Ottenio M, Tran D, Ní Annaidh A, Gilchrist MD, Bruyère K (2015). Strain rate and anisotropy effects on the tensile failure characteristics of human skin. J Mech Behav Biomed Mater.

[CR60] Pailler-Mattei C, Bec S, Zahouani H (2008). In vivo measurements of the elastic mechanical properties of human skin by indentation tests. Med Eng Phys.

[CR61] Pensalfini M, Weickenmeier J, Rominger M, Santoprete R, Distler O, Mazza E (2018). Location-specific mechanical response and morphology of facial soft tissues. J Mech Behav Biomed Mater.

[CR62] Reed RJ, Ackerman AB (1973). Pathology of the adventitial dermis: anatomic observations and biologic speculations. Hum Pathol.

[CR63] Ridge MD, Wright V (1966). The directional effects of skin. A bio-engineering study of skin with particular reference to Langer’s lines. J Invest Dermatol.

[CR64] Rohrbach DH, Timpl R (1993). Molecular and cellular aspects of basement membranes. Cell biology.

[CR65] Rubin MB, Bodner SR (2002). A three-dimensional nonlinear model for dissipative response of soft tissue. Int J Solids Struct.

[CR66] Ruth K, Freinkel M (2001) The biology of the skin. Parthenon Publishing

[CR67] Sheldon H (2011) Morphology of adipose tissue: a microscopic anatomy of fat, American Cancer Society, pp 125–139

[CR68] Smith LT, Holbrook KA, Byers PH (1982). Structure of the dermal matrix during development and in the adult. J Investig Dermatol.

[CR69] Soetens JF, van Vijven M, Bader DL, Peters GW, Oomens CW (2018). A model of human skin under large amplitude oscillatory shear. J Mech Behav Biomed Mater.

[CR70] Sommer G, Eder M, Kovacs L, Pathak H, Bonitz L, Mueller C, Regitnig P, Holzapfel G (2013). Multiaxial mechanical properties and constitutive modeling of human adipose tissue: a basis for preoperative simulations in plastic and reconstructive surgery. Acta Biomater.

[CR71] Sorrell JM, Caplan AI (2004). Fibroblast heterogeneity: more than skin deep. J Cell Sci.

[CR72] Stewart KJ (1995). A quantitative ultrastructural study of collagen fibrils in human skin normal scars, and hypertrophic scars. Clin Anat.

[CR73] Stracuzzi A, Mazza E, Ehret AE (2018). Chemomechanical models for soft tissues based on the reconciliation of porous media and swelling polymer theories. ZAMM Zeitschrift fur Angewandte Mathematik und Mechanik.

[CR74] Tonge TK, Atlan LS, Voo LM, Nguyen TD (2013). Full-field bulge test for planar anisotropic tissues: Part I—experimental methods applied to human skin tissue. Acta Biomater.

[CR75] Turcan I, Jonkman MF (2015) Blistering disease: insight from the hemidesmosome and other components of the dermal-epidermal junction. Cell Tissue Res 360 10.1007/s00441-014-2021-7 25502077

[CR76] Ueda M, Saito S, Murata T, Hirano T, Bise R, Kabashima K, Suzuki S (2019). Combined multiphoton imaging and biaxial tissue extension for quantitative analysis of geometric fiber organization in human reticular dermis. Sci Rep.

[CR77] Ventre M, Mollica F, Netti PA (2009). The effect of composition and microstructure on the viscoelastic properties of dermis. J Biomech.

[CR78] Virén T, Iivarinen JT, Sarin JK, Harvima I, Mayrovitz HN (2018). Accuracy and reliability of a hand-held in vivo skin indentation device to assess skin elasticity. Int J Cosmet Sci.

[CR79] Wahlsten A, Pensalfini M, Stracuzzi A, Restivo G, Hopf R, Mazza E (2019) On the compressibility and poroelasticity of human and murine skin. Biomech Model Mechanobiol pp 1–15 10.1007/s10237-019-01129-1 30806838

[CR80] Wang Y, Xu R, He W, Yao Z, Li H, Zhou J, Tan J, Yang S, Zhan R, Luo G, Wu J (2015). Three-dimensional histological structures of the human dermis. Tissue Eng Part C Methods.

[CR81] Weber L, Kirsch E, Müller P, Krieg T (1984). Collagen type distribution and macromolecular organization of connective tissue in different layers of human skin. J Investig Dermatol.

[CR82] Weickenmeier J, Wu R, Lecomte-Grosbras P, Witz JF, Brieu M, Winklhofer S, Andreisek G, Mazza E (2014) Experimental characterization and simulation of layer interaction in facial soft tissues. In: Cotin S (ed) Bello F. Biomedical simulation. Springer International Publishing, pp 233–241

[CR83] Weickenmeier J, Jabareen M, Mazza E (2015). Suction based mechanical characterization of superficial facial soft tissues. J Biomech.

[CR84] Wellen J, Helmer KG, Grigg P, Sotak CH (2004) Application of porous-media theory to the investigation of water ADC changes in rabbit Achilles tendon caused by tensile loading. J Magn Reson 10.1016/j.jmr.2004.04.021 15324757

[CR85] Zhao Y, Feng B, Lee J, Lu N, Pierce D (2020). A multi-layered computational model for wrinkling of human skin predicts aging effects. J Mech Behav Biomed Mater.

[CR86] Zöllner AM, Buganza Tepole A, Kuhl E (2012). On the biomechanics and mechanobiology of growing skin. J Theor Biol.

[CR87] Zöllner AM, Holland MA, Honda KS, Gosain AK, Kuhl E (2013) Growth on demand: reviewing the mechanobiology of stretched skin. J Mech Behav Biomed Mater 10.1016/j.jmbbm.2013.03.018 PMC3758413 23623569

