# Supplementary material for: A biphasic multilayer computational model of human skin
Source: Biomech Model Mechanobiol. 2021 Feb 10;20(3):969–82. doi: 10.1007/s10237-021-01424-w (PMC8154831; doi:10.1007/s10237-021-01424-w)
Supplement: Supplementary file 1 — Supplementary material 1 (pdf 638 KB) [file 10237_2021_1424_MOESM1_ESM.pdf]

Supplementary Material

A sensitivity analysis was performed to evaluate the influence of selected model parameters. These are: the multiplier of the strain energy function μ_0, the parameters governing fibers‘ nonlinearity for both elastic and dissipative element m_{4fe} and m_{4fd} and the permeability k_0. Parameters are varied individually for each layer. The tested ranges vary for each parameter: μ_0 is reduced to 0.6 and increased to 1.4 of its original values. m_{4fe} and m_{4fd} are reduced to 0.8 and increased to 1.2 of their value. Note that reducing these parameters results in a stiffer response. k_0 was changed to 0.1 and 10 times its original values. The results are shown in figures A.1 to A.3.

The very small influence of changes in μ_0 underlines that all cases analyzed result in a response well beyond the initial linear response of each tissue. Changes in the reticular dermis had the largest impact, see figure A.1 (a) and (d), but all results remains well within the scatter of the experimental data. Changes in papillary dermis and epidermis do not influence the global response (b,e,h) and (c,f,i).

Stronger effects are observed for changes in the nonlinearity parameter. As depicted in figure A.2 variation of m_{4fe} and m_{4fd} in each layer considerably affect the response. Adapting the nonlinearity in the reticular dermis strongly influences the uniaxial and the 8 mm suction response (a,d) such that both changes go beyond the experimental scatter. The influence for the 2 mm response is lower and the simulation stay well within the experimental values. Interestingly, the effects of changes of the nonlinearity parameter in the papillary dermis and the epidermis are reversed: the influence is small for uniaxial and 8 mm suction response (b,e) and (c,f), while it increases for the 2 mm response and is in the same order of magnitude as the differences observed for changes of this parameters in the reticular dermis. This again underlines the influence of the upper layers in suction experiments with small opening diameters.

A similar picture is seen for changes in the permeability depicted in figure A.3. A higher permeability softens the material response. A visible effect is observed for uniaxial and 8 mm suction only for changes in the reticular dermis (a,d). For the 2 mm response, changes in papillary dermis and epidermis do have a

visible influence (h,i), with the effect of changes in papillary dermis being strongest.

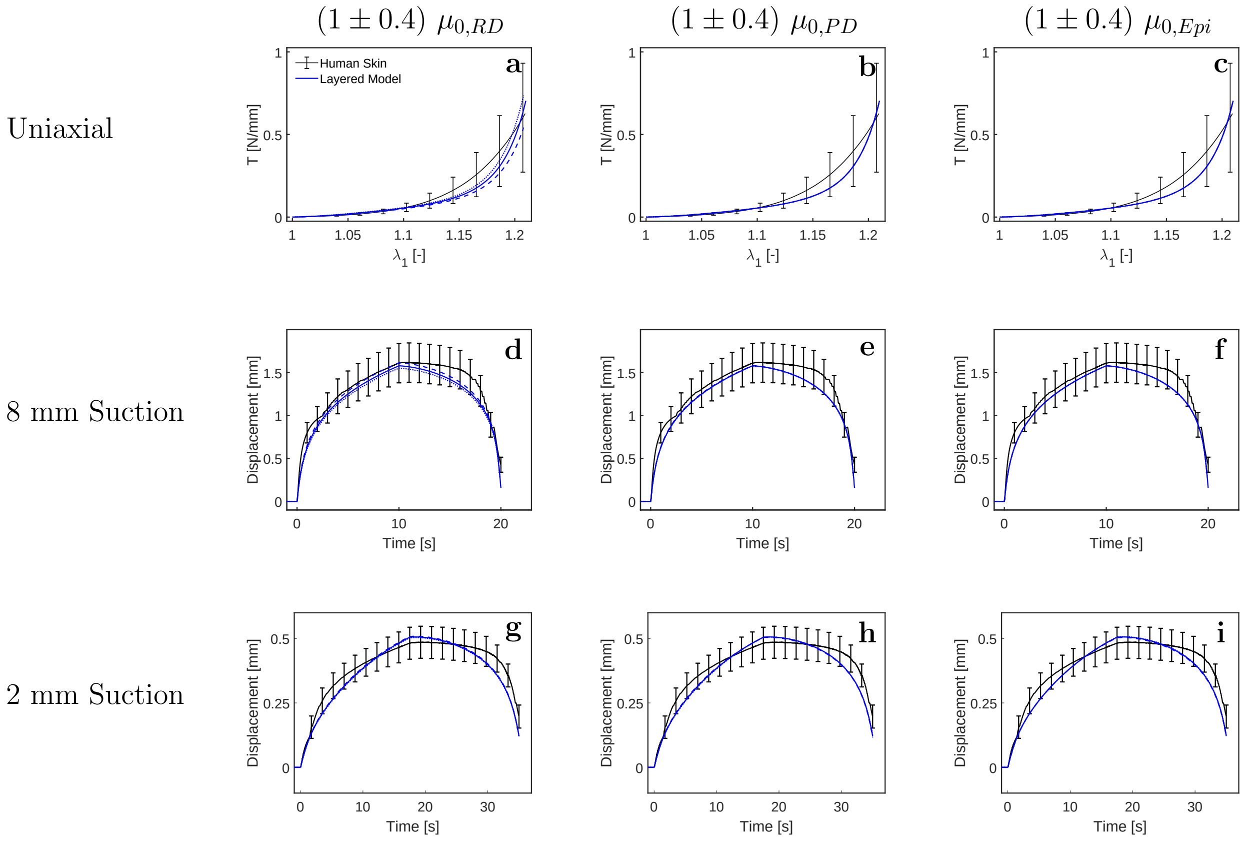

Figure A.1: Variation of the prefactor μ_0 has little influence on the overall response. Changes in the parameter to 0.6 or 1.4 of its original values in papillary dermis and epidermis result in no visible changes. Small differences are only visible for the uniaxial and the 8 mm suction simulation when changing the factor in the reticular dermis.

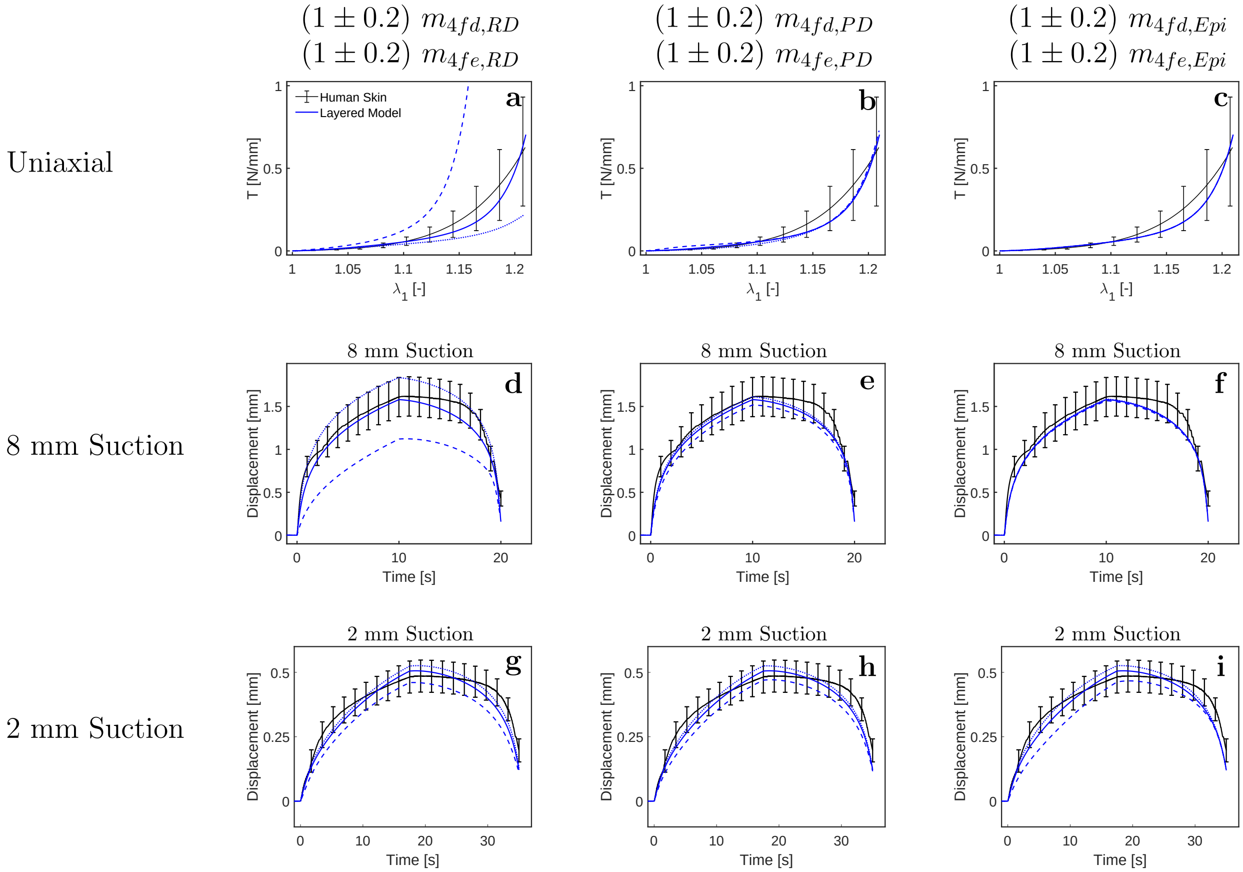

Figure A.2: Changes in the nonlinearity parameters of the fibers have significant influence on the global response. For the uniaxial simulations, only parameter variations in the reticular dermis (a) influence the result; Changes in papillary dermis (b) and epidermis (c) have negligible influence. Variation of fibers' nonlinearity in papillary dermis and epidermis have increased influence for suction experiments, with the effect being stronger for 2 mm than for 8 mm (e,h) and (f,i). The trend is opposite for the reticular dermis (d,h).

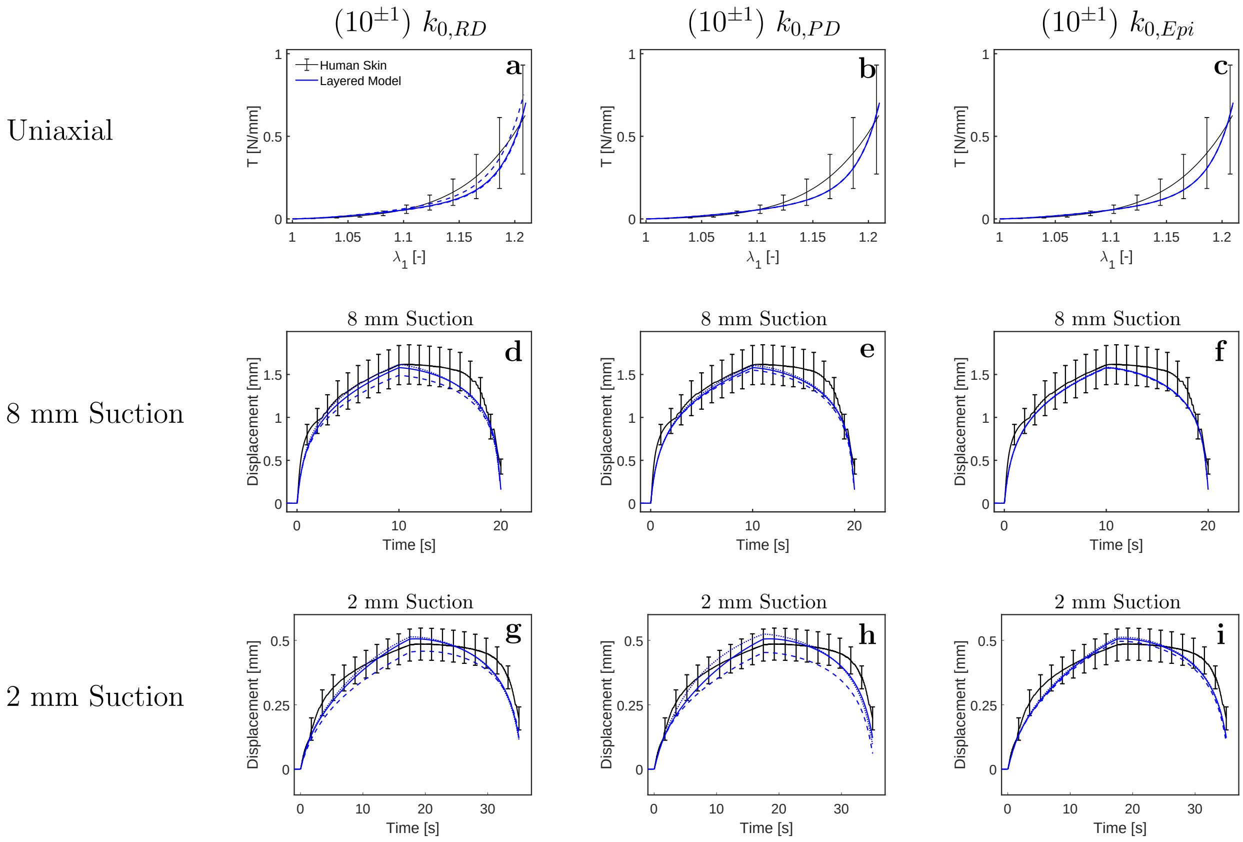

Figure A.3: Variations in permeability only modestly affect the response of the tissue. Permeability increase in the reticular dermis visibly influence the response in both uniaxial and suction response (a,d,g). Changes in papillary dermis and epidermis have negligible influence on the uniaxial response and the 8 mm suction response (b,e) and (c,f). The effect is visible for the 2 mm suction (h,i), especially for changes in the papillary dermis

In several cases, changes of parameters have only little influence on the predicted global response. This indicates that the minimization of the cost function in the inverse analysis might result in many local minima. Thus, as discussed in section 5, the selected parameter set represents a possible solution among several that might yield a prediction of similar quality.
